# Distinguishing mild cognitive impairment from healthy aging and Alzheimer’s Disease: The contribution of the INECO Frontal Screening (IFS)

**DOI:** 10.1371/journal.pone.0221873

**Published:** 2019-09-10

**Authors:** Helena S. Moreira, Ana Sofia Costa, Álvaro Machado, São Luís Castro, César F. Lima, Selene G. Vicente

**Affiliations:** 1 Centre for Psychology at University of Porto, Porto, Portugal; 2 Neurocognition Unit, Department of Neurology, Hospital de Braga, Braga, Portugal; 3 Department of Neurology, RWTH Aachen University, Aachen, Germany; 4 JARA Institute Molecular Neuroscience and Neuroimaging, Aachen, Germany; 5 Instituto Universitário de Lisboa (ISCTE-IUL), Lisboa, Portugal; Nathan S Kline Institute, UNITED STATES

## Abstract

Executive functions are affected differently in healthy aging, Mild Cognitive Impairment (MCI) and Alzheimer’s Disease (AD), and evaluating them is important for differential diagnosis. The INECO Frontal Screening (IFS) is a brief neuropsychological screening tool, developed to assess executive dysfunction in neurodegenerative disorders. Goals: We aimed to examine whether and how MCI patients can be differentiated from cognitively healthy controls (HC) and mild to moderate AD patients based on IFS performance. We also explored how IFS scores are associated with age, years of education, and depressive/anxious symptoms (as assessed by the Hospital Anxiety and Depression Scale). Method: IFS total scores were compared between 26 HC, 32 MCI and 21 mild to moderate AD patients. The three groups were matched for age and education. The Area Under the Curve (AUC) was analyzed and optimal cut-offs were determined. Results: Healthy participants had higher IFS scores than both clinical groups, and MCI patients had higher scores than AD patients. IFS showed high diagnostic accuracy for the detection of MCI (AUC = .89, *p* < .001) and AD (AUC = .99, *p* < .001), and for the differentiation between the clinical groups (AUC = .76, *p* < .001). We provide optimal cut-offs for the identification of MCI and AD and for their differentiation. We also found that, in general, higher education predicted higher IFS scores (no associations with age and depressive/anxious symptoms were observed). Altogether, these findings indicate that evaluating executive functions with the IFS can be valuable for the identification of MCI, a high-risk group for dementia, and for differentiating this condition from healthy aging and AD.

## Introduction

Executive function (EF) is an overarching term referring to the coordinated operation of specific cognitive processes (e.g., planning, working memory, self-monitoring) that direct cognition, emotion and motor activity during the accomplishment of goals, allowing individuals to respond adaptively to their environment [1]. Structural and functional neuroimaging studies [2,3] indicate that the prefrontal cortex is pivotal for EF, though other cortical (e.g., parietal areas [4]) and subcortical (e.g., cerebellum [5]) structures are also involved.

EF seems to follow an inverted U trajectory across the lifespan, declining in late adult years [6,7]. Cross-sectional [8] and longitudinal [9] studies show that EF are the first cognitive functions to decline with aging, and the magnitude of this decline is greater than the one observed for cognitive domains such as episodic memory, reasoning, and spatial abilities [7]. Working memory [10], inhibition, and planning [11,12] are the most affected EF processes, accounting for variance in cognitive domains such as learning and episodic memory [13,14]. This age-related decline in EF has been associated with structural and functional changes in frontal lobe areas [9,15,16]. Importantly, such decline is generally subtle and does not compromise autonomy. When it does, there is a risk of association with neurodegenerative disorders.

Mild Cognitive Impairment (MCI) represents a stage of cognitive function between the expected decline seen in healthy aging and that seen in dementia, i.e., individuals with MCI have a more pronounced cognitive impairment than what would be expected for their age and education, but do not meet functional criteria for dementia [17,18]. Longitudinal studies indicate higher rates of progression to dementia in MCI patients vs. healthy controls [19,20], a finding highlighting the predictive value of this clinical entity. There is considerable heterogeneity regarding the cause and prognosis of MCI. Most often, patients develop Alzheimer’s dementia (AD) [21]. However, MCI can also result from other degenerative conditions such as Parkinson’s disease [22] and frontotemporal lobar degeneration [23], or non-degenerative disorders such as cerebrovascular pathologies [24], brain tumors and medication [21]. Based on clinical presentation, MCI has been classified into four subtypes: amnestic MCI single domain, amnestic MCI multiple-domain, non-amnestic MCI single domain, and non-amnestic MCI multiple-domain [18,25]. Deficits in EF are common in amnestic multiple domain and non-amnestic single or multiple-domain MCI, with patients performing worse than HC in EF tasks [10,26]. These impairments affect patients’ functionality [27] and are associated with depressive and anxious symptoms [28]. Furthermore, MCI patients who present with executive and memory deficits combined are at a particularly high risk of progression to dementia (as compared to single domain amnestic or dysexecutive MCI) [29]. However, the study of EF in MCI remains a relatively poorly explored topic, particularly in what respects to comparisons with AD patients. Such studies are important for a better understanding of executive impairments in different conditions, and they can also contribute to better differential diagnosis.

AD is typically characterized by impairments in episodic memory [30,31], but discernable deficits in EF are also often present in components such as verbal inhibition [32,33], planning [12], abstraction [30] and working memory [34]. Executive dysfunction in AD patients is associated with increased frontoparietal brain activity in relation to task demands in functional MRI and positron emission tomography studies [35]. Deficits in EF impact on AD patients’ daily life, being associated with functional disability, with a greater need for care [36], and with the emergence of neuropsychiatric symptoms such as agitation, disinhibition [37], and psychotic symptoms [38].

The INECO Frontal Screening (IFS) [39] is a brief screening tool that assesses three executive domains (response inhibition and set shifting, abstraction, and working memory) and presents good psychometric properties (for a review, see Moreira and colleagues [40]). The IFS can discriminate controls from patients with frontotemporal dementia (FTD) [39,41,42] and AD [33,39,42], and it can also discriminate between some clinical groups, with FTD patients scoring lower than those with major depression [41] and AD [39,43]. To our knowledge, though, the utility of this tool in the context of MCI remains unknown. Previous studies indicate that the Montreal Cognitive Assessment (MoCA) [44], a screening tool for global cognition, can distinguish MCI from HC and early stage AD patients [45,46]. Can EF, as assessed with the IFS, also distinguish MCI from the other groups? Another relevant issue concerns the potential effects of age and education on IFS performance, effects that might have implications for the use of this tool in MCI and in clinical practice more broadly. A number of studies report positive effects of education [33,47,48], but as for age results are mixed: some studies report negative effects of age on IFS performance [33], while others do not [47,48].

The goal of the current study was to investigate whether MCI patients can be differentiated from healthy controls and AD patients based on EF abilities, using IFS. The contribution of each IFS subtest was also analyzed, and optimal cut-off points to detect MCI and AD, as well as to differentiate these two clinical groups, were established. We hypothesized that MCI patients would present lower performance than HC on EF, as indexed by lower IFS scores, but higher performance than AD patients. Additionally, the influence of age, years of education and depressive/anxious symptomatology (as indexed by the Hospital Anxiety and Depression Scale; HADS) [49] on IFS performance was examined. We expected IFS performance to be negatively influenced by age and depressive/anxious symptoms and positively influenced by years of education.

## Materials and methods

### Participants

A total of 79 participants were included in this study: 26 HC, 32 MCI patients and 21 AD patients. Patients were retrospectively selected from a specialized memory outpatient clinic, and had their baseline neurological and neuropsychological assessment between 2013 and 2015 (all the results reported here are based on this initial baseline assessment). The Ethics Committee for Health of Hospital de Braga (Comissão de Ética para a Saúde Hospital de Braga) approved the retrospective analysis of the patients’ clinical information (CESHB 054/2016) and the study was conducted in agreement with the Helsinki Declaration. All data were anonymized before being accessed for research purposes. We were not required by the ethics committee to obtain informed consent from the patients, as the clinical data were entirely extracted from the available clinical records, i.e., no additional tests were specifically administered for the current study. This is in agreement with the Portuguese law N°. 12/2005 of 26 January. Written informed consent was obtained from all HC participants.

Patients were consecutively included in the study if they had (1) criteria for MCI or AD, and (2) available information regarding the MMSE, MoCA, HADS and IFS total and subtest scores. They were classified as MCI using Petersen and colleagues [21] revised criteria: (1) objective impairment in formal neuropsychological measures (total score on MMSE and MoCA at least 1.5 SD below the demographically corrected mean) [50,45] and (2) preserved activities of daily living. According to the standardized neuropsychological assessment, 19 MCI patients were classified as “amnestic MCI” (multiple-domain) and 13 as “non-amnestic MCI” (single or multiple-domain). For each patient, the probable etiology of MCI was determined by an experienced neurologist, based on detailed diagnostic work-up (including clinical history and neurological, neuropsychological, and functional and/or structural neuroimaging examinations). This information pointed to potential neurodegenerative pathology in most cases. Cases with clinical records of primary psychiatric disease, significant vascular pathology and other focal lesions (e.g., traumatic brain injury) were excluded. Diagnosis of AD was based on standard diagnostic procedures, according to national and international diagnostic guidelines for probable AD [51], and included the clinical history, neurological examination, routine blood tests, clinical imaging of the brain (computed tomography or magnetic resonance), and a comprehensive neuropsychological assessment (for protocol details see Costa et al. [52]). In the present study, the IFS was not considered for diagnostic purposes. For some patients, we also had information on cerebrospinal fluid biomarkers of AD (AΒ, total tau, and phospho-tau, AΒ/tau ratio) and/or functional imaging (fludeoxyglucose F18 positron emission tomography or single-photon emission computed tomography). Most patients were over 65-years-old when diagnosed, and therefore potentially reflect sporadic rather than early-onset familial cases of AD [53]; only four of them were younger, but none of these had familial history of the disease and/or mutations in genes APOɛ4, PSEN1, PSEN 2 and APP. Only patients with mild-to-moderate dementia severity were included (stages 4 and 5 of Global Deterioration Scale) [54], all of them with MMSE scores above 10 (mild and moderate AD stage) [55].

The presence of depressive symptoms was not an exclusion criterion, given its prodromal value in MCI [56,57] and links with neuropathologic processes of AD [58]. However, in general, the magnitude of these symptoms was similar across groups (*p* = .79, see Table 1).

**Table 1:** Demographic and neuropsychological characteristics of Healthy Controls (HC), Mild Cognitive Impairment (MCI) and Alzheimer’s Disease (AD) participants.

	HC (n = 26)		MCI patients (n = 32)		AD patients (n = 21)		
	M (SD)	Range	M (SD)	Range	M (SD)	Range	*p*
**Age** **(years)**	68.42 (8.39)	58–83	68.03 (7.29)	55–81	72.48 (9.16)	55–85	.13
**Education** **(years)**	5.88 (1.99)	3–9	6.53 (3.69)	4–16	5.48 (2.77)	3–13	.85
**Sex (M:F)**	10:16	-	17:15	-	8:13	-	.43
**MMSE (/30)**	29.62 (0.70)^+^^,^^++^	27–30	27.69 (1.31) ^++^^,^ ^+++^	26–30	20.52 (3.42) ^+^^,^ ^+++^	14–26	**< .001**
**MoCA (/30)**	25.27 (2.44) ^+^^,^^++^	19–30	18.00 (4.21) ^++^^,^ ^+++^	8–28	11.89 (3.70) ^+^^,^ ^+++^	6–19	**< .001**
**HADS (/21)**	4.15 (5.99)	0–23	3.58 (3.53)	0–12	4.71 (4.47)	0–16	.79

M, male; F, Female; MMSE, Mini Mental State Examination; MoCA, Montreal Cognitive Assessment; HADS, Hospital Anxiety and Depression Scale.

^+^ Differ from MCI (*p* < .001).

^++^ Differ from AD (*p* < .001).

^+++^ Differ from HC (*p* < .001).

HC participants were volunteers recruited in the community and they were matched with the MCI and AD patients for age and education. All of them were autonomous and had no known history of alcoholism/substance abuse, brain injury, neurological/psychiatric conditions, or other significant medical conditions that could affect cognition. For inclusion, normal performance on MMSE and MoCA, as indicated by scores above the age and education corrected cut-offs, was required [50, 45].

### Neuropsychological assessment

The sociodemographic and clinical data were collected through a clinical interview or obtained from patient records. The MMSE and MoCA were used as measures of global cognition. Symptoms of depression and anxiety were assessed with the HADS. All participants completed the Portuguese version of IFS [33]. The IFS takes around 10 minutes to administer and comprises eight subtests that cover three domains (response inhibition and set shifting, abstraction, and working memory), and inspect the following specific processes: motor programming (Luria’s motor series fist, edge, palm), sensitivity to interference (conflicting instructions), inhibitory control (Go/no Go), verbal inhibitory control (modified Hayling test), abstraction capacity (proverb interpretation), working memory for digits (backward digit span), verbal working memory (months of the year backward), and spatial working memory (modified Corsi block tapping test). The tasks motor series, conflicting instructions, and Go/no Go were taken from Frontal Assessment Battery (FAB) [59], a widely used screening test of EF. The other ones are specific to the IFS and were selected to optimize the sensitivity of this tool in comparison to the FAB. More comparative research is needed, but the FAB was found to discriminate between controls and AD patients less well than IFS (for a review see Moreira et al., [40]). IFS total score ranges between 0 and 30, with lower scores suggesting worse executive functioning.

### Statistical analysis

Descriptive statistics were used for sample characterization. A multiple linear regression (enter method) was performed to examine the extent to which sociodemographic variables (education and age) and affective symptoms (anxious/depressive) influence IFS total score, even when the cognitive impairment associated to each diagnostic group is accounted for (i.e., Group was also included in the model). Comparisons between the three groups in IFS total and subtests’ scores were explored with one-way ANOVAs, and follow-up paired comparisons were Bonferroni corrected. Values of skewness and kurtosis were always below 2 and 7, respectively (skewness, range = -1.38–1.30; kurtosis, range = -1.19–1.14), suggesting that there is no large departure from normality in the data [60]. The diagnostic accuracy of the IFS total score for the detection of MCI and AD patients was assessed with receiver operation characteristics (ROC) curve analyses. The optimal cut-offs scores were selected based on the Youden index, with higher values indicating best sensitivity (the proportion of participants with cognitive impairment correctly identified as such) and specificity (the proportion of participants without cognitive impairment correctly identified as such). Finally, a discriminant analysis was carried out to determine the contribution of each IFS subtest for the group classification. All effects were considered significant at *p* < .05. Analysis were done using IBM SPSS® software (version 24).

## Results

### Sample characterization and influence of age, education, depressive/anxious symptoms on IFS scores

The characteristics of HC participants, MCI and AD patients are summarized in Table 1. The three groups did not differ in age [*F* (2,78) = 2.12, *p =* .13, η_p_^2^ = .05], years of education [*F* (2,78) = 0.85, *p =* .43, η_p_^2^ = .02), sex [χ^2^ (2) = 1.7, *p* = .43] and in HADS total score [*F* (2,61) = 0.23, *p =* .79, η_p_^2^ = .01]. Note that the education level of our sample is lower than the observed in other similar studies [39,42,43], but it is representative of the majority of Portuguese elderly population. According to Censos 2011 [61], the majority of people over 65 years in Portugal completed only 4 years of formal education, corresponding to 1st Cycle of Basic Education (67.27%, 62.65%, and 56.80% of the people aged 65–69 years, 70–74 years, and 75–79 years, respectively).

MMSE scores significantly differed between the three groups, as expected [*F* (2,78) = 133.38, *p <* .001, η_p_^2^ = .78]; they were lower in the AD group than in both MCI (*p* < .001) and HC (*p* < .001) groups, and MCI patients also underperformed HC participants (*p* = .001). Please note that for MMSE there was a very low range in HC (between 27–30), reinforcing the well documented celling effect of this measure [62]. Significant differences between groups were also found for MoCA total scores [*F* (2,75) = 76.84, *p <* .001, η_p_^2^ = .68]: the AD group presented the lowest scores, followed by MCI and HC groups, that also significantly differed from each other (all *p*s < .001).

A multiple regression analysis was conducted with group, age, years of education and HADS total score as independent variables and IFS total score as dependent variable. The resulting model explained 73% of the IFS variance [adjusted *R*^2^ = 0.73, *F* (4,61) = 41.13, *p* < .001]. Group was the strongest predictor (ß = 0.76, t = 11.19, *p* < .001), but education also significantly predicted unique variance in IFS performance (ß = 0.37, t = 5.22, *p* < .001), with more years of education being associated with better results in this tool. Neither age nor HADS total score were significant predictors (*p =* .67 and *p =* .83, respectively).

### Sensitivity of the IFS to differentiate MCI patients from HC and AD patients

**Fig 1:**
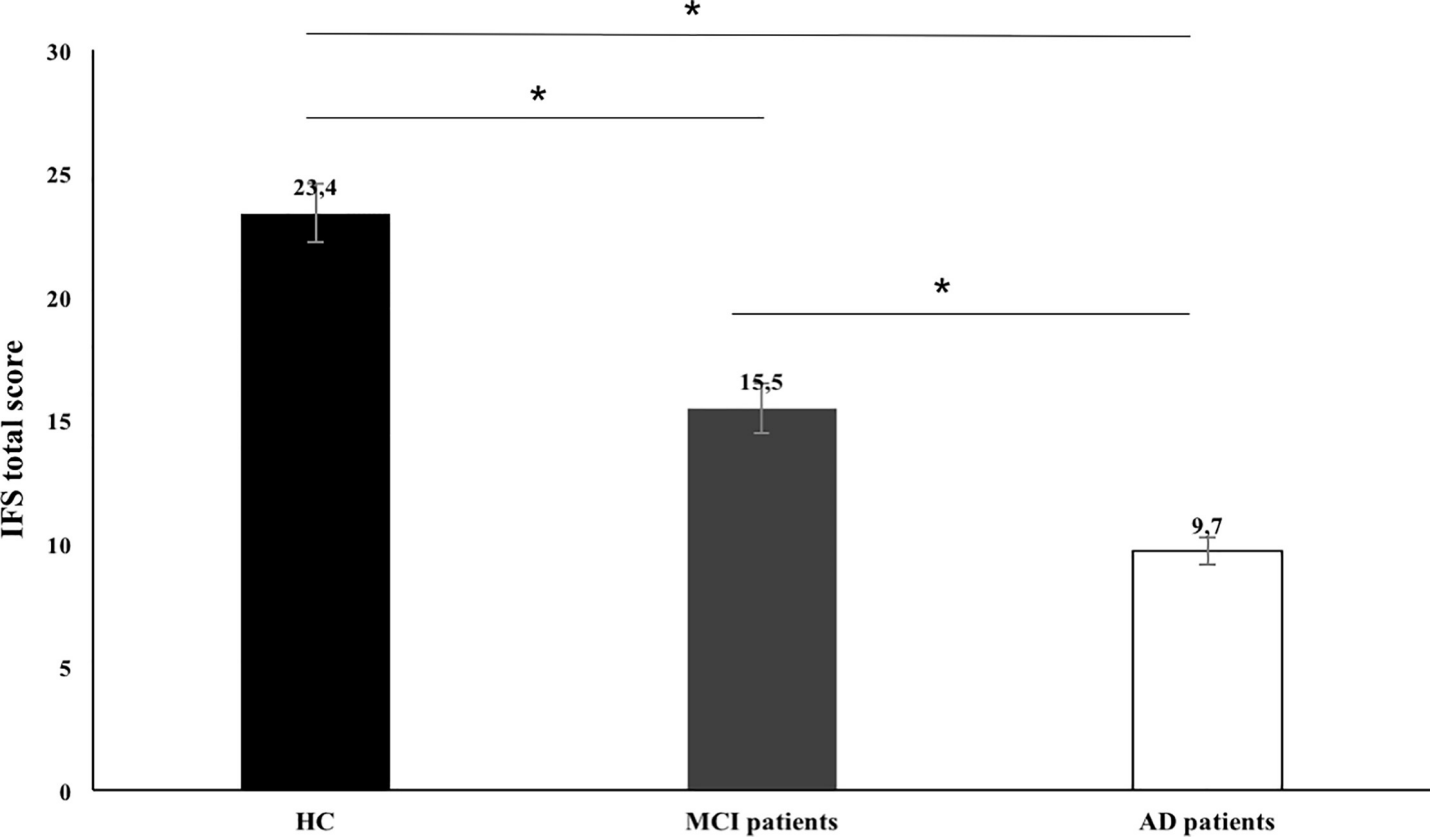
Total IFS total scores for control and clinical groups. HC, Healthy Controls; MCI, patients with Mild Cognitive Impairment (MCI); AD, patients with Alzheimer’s dementia. Error bars represent standard error. * *p* < .05.

The performance of HC participants, MCI and AD patients on the IFS is depicted in Fig 1. As hypothesized, significant differences were found between the three groups [*F* (2, 78) = 47.57, *p* < .001, η_p_^2^ = 0.56]. Specifically, MCI patients (*M* = 15.47; *SD* = 5.63; range = 4–27) had lower total scores than HC (*M* = 23.44; *SD* = 2.86; range = 16.5–29; *p* < .001) but higher than AD patients (*M* = 9.74; *SD* = 5.52; range = 3–20; *p* < .001). In order to determine if these differences remain significant after controlling for variables that we have shown to predict IFS performance (years of education), we conducted an analysis of covariance (ANCOVA). The group effect was confirmed even when this confounding variable was controlled [*F* (2,75) = 14. 58, *p* < .001, η_p_^2^ = .67], between all of the three groups: MCI patients vs. HC participants (*p* < .001), MCI patients vs. AD patients (*p* < .001) and AD vs. HC participants (*p* < .001). The same results were obtained when the ANCOVA also included sex as a covariate.

Since there were two identifiable subtypes within our sample of MCI participants (amnestic and non-amnestic), we also conducted an additional exploratory analysis to check if there were discernible differences between them in IFS total and subtests scores. No differences were found in IFS total score (*p* = .23) or subscores (*p*s > .74), apart from a marginally significant effect in the Conflicting Instructions subtest [with amnestic MCI performing slightly higher than non-amnestic, *t*(17.62) = 2.10, *p* = .05].

**Fig 2:**
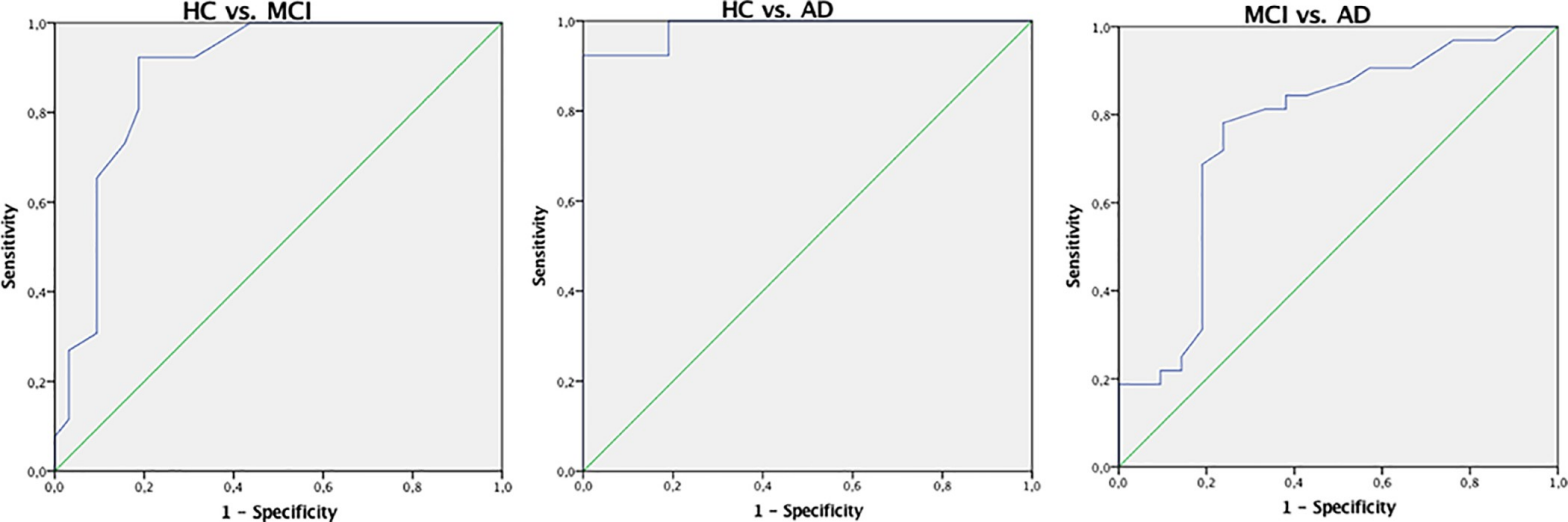
Receiver operating characteristic (ROC) curves of the IFS in the differentiation between control and clinical groups, as well as between the two clinical groups. HC from MCI patients (left) and AD patients (center); MCI from AD patients (right).

ROC curve analyses were carried out to evaluate the diagnostic accuracy of the IFS (see Fig 2). Regarding the discrimination between HC and MCI participants, this analysis generated an optimal cut-off of 20 points, with 92% sensitivity and 81% specificity (AUC of 0.89, CI = 0.80–0. 98). In the discrimination between HC and AD participants, the optimal cut-off was 15 points, with 100% sensitivity of and 81% specificity (AUC of 0.99, CI = 0.96–1.00). A cut-off of 12.5 points reached the best sensitivity (78%) and specificity (76%) in the differentiation between clinical groups of MCI and AD patients (AUC of 0.76, CI = 0.62–0.90).

To examine the performance of the three groups in specific executive domains, the scores of each IFS subtest were compared (see Table 2). The effect of group was largest for the Hayling test [*F* (2,76) = 44.58, *p* < .001, η _p_^2^ = .54], Proverbs interpretation [*F* (2,76) = 31.89, *p* < .001, η_p_^2^ = .46], Backward digit span [*F* (2,76) = 23.94, *p* < .001, η_p_^2^ = .39], and Motor series [*F* (2,76) = 19.74, η_p_^2^ = .34], suggesting that these were the subtests that were more sensitive to general variation across groups. For the Hayling test, Proverbs interpretation, and Backward digit span, AD and MCI patients scored lower than HC, but no significant differences were found between AD and MCI patients (Hayling test, HC vs. AD, *p* < .001; HC vs. MCI, *p* < .001 and MCI vs. AD, *p* = .66; Proverbs interpretation, HC vs. AD, *p* < .001; HC vs. MCI, *p* < .001 and MCI vs. AD, *p* = .26; Backward digit span, HC vs. AD, *p* < .001; HC vs. MCI, *p* < .001 and MCI vs. AD, *p* = .79). For the Motor series, significant differences were obtained between the three groups, with AD patients presenting the worst results, followed by MCI patients and by the HC (HC vs. AD, *p* < .001; HC vs. MCI, *p* < .001 and MCI vs. AD, *p* = .05). The remaining four subtests (Conflicting instructions, Go/no go, Verbal working memory and Spatial working memory) were not sensitive to differences between HC and MCI patients, but they discriminated between HC and AD patients, and between MCI and AD patients (Conflicting instructions: *F* (2,76) = 11.41, *p* < .001, η_p_^2^ = .21; HC vs. AD, *p* <. 001; HC vs. MCI, *p* = .81 and MCI vs. AD, *p* = .001; Go /no go: *F* (2,76) = 9.03, *p* < .001; η_p_^2^ = .19; HC vs. AD, *p* < .001; HC vs. MCI, *p* = 1.00 and MCI vs. AD, *p* = .003; Verbal working memory: *F* (2,76) = 16.32, *p* < .001, η_p_^2^ = .30; HC vs. AD, *p* < .001; HC vs. MCI, *p* = .21 and MCI vs. AD, *p* < .001; Spatial working memory: *F* (2,76) = 12.19, *p* < .001, η_p_^2^ = .24; HC vs. AD, *p* < .001; HC vs. MCI, *p* = .06 and MCI vs. AD, *p* = .02).

**Table 2:** Scores in IFS subtests of Healthy Controls (HC), Mild Cognitive Impairment (MCI) patients and Alzheimer’s Disease (AD) patients.

	HC (*n* = 26)	MCI patients (*n* = 32)	AD patients (*n* = 21)	*p*	Post Hoc comparisons
IFS subtests	M (SD)	M (SD)	M (SD)		
**Motor series**	2.92 (0.39)	1.97 (1.03)	1.38 (0.97)	< .001	HC > MCI > AD
**Conflicting instructions**	2.69 (0.47)	2.41 (1.04)	1.38 (1.28)	< .001	HC >AD; MCI > AD; HC = MCI
**Go/ no go**	2.54 (0.71)	2.31 (1.06)	1.29 (1.38)	< .001	HC >AD; MCI > AD; HC = MCI
**Backward digit span**	3.54 (1.00)	2.06 (0.98)	1.76 (0.83)	< .001	HC >AD; HC > MCI; MCI = AD
**Verbal working memory**	1.92 (0.27)	1.59 (0.71)	0.81 (0.93)	< .001	HC >AD; MCI > AD; HC = MCI
**Spatial working memory**	2.54 (0.81)	1.97 (1.09)	1.25 (0.63)	< .001	HC >AD; MCI > AD; HC = MCI
**Proverbs**	2.21 (0.64)	0.92 (0.92)	0.55 (0.67)	< .001	HC >AD; HC > MCI; MCI = AD
**Hayling Test**	5.15 (1.12)	1.88 (1.79)	1.33 (1.62)	< .001	HC >AD; HC > MCI; MCI = AD

IFS, INECO Frontal Screening

Finally, a discriminant analysis (enter method) was conducted to further understand how IFS scores could be informative about differences between groups. The discriminant function was able to correctly classify the group membership of 79% of the patients (Wilks' Lambda = 0.25; χ^*2*^ (16) = 98.77, *p* < .001). The three subtests that presented the highest contribution were: 81% in the Hayling test, 69% in the Proverb interpretation, and 63% in the Backward digit span. Note that these were the same subtests that showed larger effect sizes in the previously presented comparisons across groups.

## Discussion

EF are higher-order processes that are differentially compromised in healthy aging, MCI and AD. In previous studies, the IFS [39] has shown good discriminant accuracy between healthy participants and patients with dementia, and also between patients with different types of dementia. Nonetheless, its utility in MCI–a stage of cognitive decline that might progress to dementia—had not yet been explored. The present study provides four main new findings. First, MCI patients can be distinguished from healthy participants and early to moderate AD patients based on the IFS total score. Specifically, the MCI group had lower scores than controls and higher scores than patients with AD. Second, IFS performance was not influenced by age or depressive/anxious symptoms but was positively influenced by years of education. Nonetheless, its discriminative power in MCI vs. HC, MCI vs. AD and MCI vs. AD remains significant even when controlling for education. Third, cut-off scores of 20 and 12.5 provided optimal sensitivity and specificity in the identification of MCI vs. controls and of MCI vs. AD, respectively. Fourth, performance on IFS subtests can also be informative to differentiate between healthy elderlies, MCI and AD patients. These conclusions are discussed in detail below.

The IFS total score accurately distinguished MCI patients from HC and AD patients. Finding that the IFS differentiates AD from HC adds to a number of previous studies, and highlights again the utility of this screening tool in the context of this condition [33,39,42,43]. However, the current study is the first to provide evidence for the utility of IFS in the context of MCI: such utility is not only seen in analyses of score differences between groups, but also in subtests scores, discriminant analysis and analyses of diagnostic accuracy (AUC = .89 for MCI vs. HC, and AUC = .76 for MCI vs. AD). The existence of executive impairments in MCI has been documented before [26,63], but evidence for the utility of brief screening tools to assess them is limited. This finding is valuable both for research and clinical purposes, for instance for the neuropsychological characterization and newly diagnosed MCI patients (and monitoring of progression), but also for differential diagnosis (between MCI and AD). Nonetheless, we should note that AUC values were higher between MCI and HC than between MCI and AD, suggesting that the IFS might be particularly useful for the early detection of impairments in people at risk of developing dementia (and relatively less for the differentiation between MCI and AD patients). We found that a IFS cut-off of 20 reaches high values of sensitivity (.92) and specificity (.81) in the discrimination between MCI and HC. Such sensitivity and specificity values are comparable to those presented by MoCA [45], a well-established tool specifically developed for this purpose.

An analysis of subtests scores showed that these can also be informative. Subtests of verbal inhibitory control (Hayling subtest), abstraction (Proverbs), and verbal working memory (Backward digit span) were the most informative for differences between HC and MCI. However, they were less sensitive to the differences between MCI and AD, i.e., between stages of subtle cognitive impairment and dementia. The Motor series subtest, in turn, was able to discriminate between the three groups. Tasks which explore sensitivity to interference (Conflicting instructions), inhibitory control (Go no-go) and working memory (verbal and spatial working memory subtests) were more valuable for the differentiation between MCI and AD, presenting relatively lower potential for the identification of subtle deficits of MCI in comparison with HC. Thus, albeit the information provided by the IFS does not replace medical, comprehensive neuropsychological and neuroimaging techniques, the examination of total and subtests scores produces relevant information that can significantly contribute to the differential diagnosis between HC, MCI and AD patients. Which subtest(s) might be more informative will depend on the question being asked (e.g., discrimination between HC and MCI, or discrimination between HC and AD).

Contrary to our hypothesis, age did not predict the performance in IFS. This is not in line with the previous study of Moreira and colleagues [33], with a Portuguese normative sample, but is consistent with the recent study of Sanjurjo et al. [48] that aimed to evaluate the impact of demographic variables on IFS performance of healthy participants. Nonsignificant correlations between age and IFS total score were also found in the study of Ihnen and colleagues [47] with a dementia group. The absence of an age effect in IFS has been previously argued to reflect a celling effect of the measure [48], sometimes observed in the context of short screening tools [64]. Nonetheless, studies with other classic executive measures such as task-switching [8,65], verbal fluency, and the Wisconsin Card Sorting Test-Modified [66] also reported limited age effects. We should note, however, that the age range in the current study is somewhat limited, for instance in comparison to the age range of a previous study by our group in which age effects on the IFS were found [33]. Moreover, our sample is relatively small and all participants were included in the multiple regression analysis regardless of their diagnostic group (instead of including only the control ones)–this was done to increase statistical power, but it might have slightly biased the results. Thus, this question remains open and calls for more systematic research in the future. Intra-individual longitudinal analysis over time, for example, would be a more informative and stronger test to the influence of age on IFS performance. Education, in turn, significantly accounts for IFS performance, with more formal years of education being associated with higher scores. This effect has been consistently reported in previous studies with the IFS [33,47,48] but also with other executive screening measures such as the FAB [67]. Therefore, our study underlines the importance of considering education in the interpretation of the performance in executive tools [68]. Higher education levels seem to increase the contact with the evaluation contexts and to contribute to cognitive reserve, exerting a protective effect on the decline associated with healthy aging in EF [69]. Importantly, despite the influence of education, the discriminative power of this tool in the detection of EF deficits in MCI and in their differentiation from those of AD patients remains after accounting for this variable.

In an exploratory analysis, we compared the IFS total scores of patients with different MCI subtypes and found that they did not differ significantly. This is consistent with previous studies that suggest that both amnestic multiple-domain and non-amnestic single and multiple-domain MCI subtypes presented executive deficits [26,29]. However, our groups were relatively small (n = 19 for amnestic MCI and n = 13 for non-amnestic MCI) and more studies with bigger samples will be essential to provide more reliable conclusions. Another issue that deserves more attention in future studies is the relationship between the IFS and measures of functional ability. It was established that performance in instrumental activities of daily living is highly related with EF [70]. Hence, it would be expected that performance in IFS could predict the scores on functionality measures. This information is even more relevant since the differential diagnosis between MCI and dementia relies primarily in the decline in functionality [18,70].

Some limitations of our study should be acknowledged. In addition to the relatively small sample size, we did not have access to patients’ longitudinal data. Such information would be valuable particularly for MCI patients, not only to better clarify the aetiology of their cognitive impairment, but also to examine how the impairment changes over time and the rates of progression to dementia. In future work it will be important to determine the extent to which brief tools such as the IFS can provide useful information to predict cognitive and functional outcomes of MCI patients over time.

## Conclusion

This study produced evidence regarding the utility of the IFS for the identification of MCI, a well-established condition associated with a higher risk of progression to dementia. The early detection of this condition through a brief screening test enables prompting implementation of intervention strategies and managing of risk factors, thus holding potential to prevent a faster progression. Likewise, we found that performance in IFS can discriminate not only between normal aging and MCI and AD but also between these two clinical groups. The contribution of this tool for the differential diagnosis of these groups might be helpful in clinic settings where they are increasingly prevalent. Finally, our results underline the need of normative data from different levels of education, in order to avoid biased interpretations.

## Supporting information

S1 Dataset Minimal anonymized dataset. (PDF)
