# Supplementary material for: Distinguishing mild cognitive impairment from healthy aging and Alzheimer’s Disease: The contribution of the INECO Frontal Screening (IFS)
Source: PLoS One. 2019 Sep 10;14(9):e0221873. doi: 10.1371/journal.pone.0221873 (PMC6736301; doi:10.1371/journal.pone.0221873)
Supplement: S1 Dataset — (PDF) [file pone.0221873.s001.pdf]

Group	Sex	Educatio	MMSE_tota	MoCA_tota	IFS_total	IFS_MS	IFS_CI	IFS_GNG	IFS_BDS
1	M	9	26	18	20,0	3	3	3	2
1	M	4	20	12	8,0	1	0	0	3
1	F	3	20	9	5,0	1	0	0	2
1	F	9	21	19	19,0	3	2	3	3
1	M	4	22	12	6,5	1	2	0	2
1	F	3	19	13	7,5	1	0	2	2
1	F	9	14	8	5,0	2	0	0	2
1	F	4	25	11	17,5	1	2	3	2
1	M	4	24	14	7,0	2	2	1	0
1	M	9	24	15	12,0	1	0	3	3
1	F	5	16	99	8,0	2	2	0	1
1	F	13	23	17	20,0	2	3	3	3
1	F	4	23	13	10,0	1	2	0	2
1	F	6	19	12	12,0	2	3	3	2
1	M	4	14	99	3,0	0	2	0	1
1	F	3	19	99	4,0	0	0	2	1
1	M	4	23	7	13,5	2	3	3	1
1	F	7	17	9	6,0	0	0	0	1
1	F	3	23	9	6,0	1	0	1	1
1	F	4	21	10	11,5	3	3	0	2
1	M	4	18	6	3,0	0	0	0	1
2	M	9	29	23	22,0	2	3	3	4
2	M	4	28	14	11,0	2	0	3	2
2	M	4	26	19	17,5	2	3	2	3
2	F	4	29	16	16,0	2	2	3	2
2	M	9	30	28	25,0	3	3	3	4
2	F	4	26	13	4,0	2	0	0	0
2	M	4	28	22	14,0	3	3	0	2
2	M	4	27	19	12,0	3	3	0	1
2	M	4	27	17	13,0	2	2	3	2
2	M	4	29	13	14,5	0	3	3	2
2	F	4	26	19	8,0	1	2	2	2
2	F	4	26	15	14,0	3	3	2	2
2	M	9	26	17	18,0	3	3	3	2
2	F	4	27	19	7,5	0	2	2	1
2	M	4	28	19	15,5	3	3	3	2
2	F	4	26	8	15,0	3	3	3	1
2	F	11	28	18	22,5	2	3	3	3
2	M	4	27	20	17,0	3	3	3	3
2	M	4	29	13	6,0	0	0	1	2
2	F	9	27	13	25,0	0	3	0	2
2	F	4	30	21	17,0	2	3	3	2
2	F	6	27	14	14,5	2	3	3	1
2	M	12	28	20	16,5	2	3	3	3
2	M	4	27	22	13,5	1	3	3	1
2	F	4	27	17	17,5	2	3	2	2
2	F	10	27	15	14,0	3	0	2	3

2	M	14	28	24	27,0	3	3	3	4
2	F	13	29	21	19,5	2	3	3	2
2	M	4	29	20	13,0	2	3	3	1
2	F	4	26	13	6,0	0	1	1	1
2	F	11	30	20	16,5	2	2	3	1
2	M	16	29	24	22,5	3	3	3	3
3	M	4	29	21	24,0	3	2	2	4
3	F	9	29	25	24,0	3	3	3	2
3	F	4	30	23	23,0	3	2	2	3
3	F	6	30	30	26,5	3	3	3	4
3	F	9	30	27	29,0	3	3	3	6
3	M	6	30	26	27,5	3	3	3	4
3	M	6	30	25	27,0	3	3	3	5
3	F	9	30	25	22,5	3	3	3	2
3	F	4	30	24	22,0	3	2	2	4
3	M	9	30	24	23,0	3	3	3	2
3	M	9	30	25	23,0	3	3	3	4
3	F	9	30	27	26,0	3	3	3	4
3	M	4	30	25	21,0	3	2	2	3
3	F	4	30	28	23,5	3	2	2	4
3	F	4	30	26	21,0	3	2	2	3
3	M	6	30	28	24,0	3	3	3	4
3	M	6	30	28	26,0	3	3	3	4
3	F	6	30	27	23,5	3	3	2	4
3	M	4	30	23	23,5	3	3	2	3
3	F	4	30	19	21,0	3	2	2	2
3	F	6	29	24	22,0	3	3	3	2
3	F	6	29	27	25,0	3	3	3	4
3	F	3	29	25	16,5	3	3	0	4
3	F	6	29	25	25,5	3	3	3	4
3	F	4	27	22	17,0	3	2	3	3
3	M	6	29	28	22,5	1	3	3	4

1 -AD
2-MCI
3-HC

IFS_VWM	IFS_SWM	IFS_P	IFS_H	HADS_total
2	2	1,00	4	4
0	3	0,00	1	4
1	1	0,00	0	4
2	2	0,00	4	12
0	1	0,50	0	5
0	1	1,50	0	4
0	1	0,00	0	4
2	1	1,50	5	4
0	1	1,00	0	0
2	1	0,00	2	
0	1	0,00	2	2
2	1	2,00	4	12
2	1	1,00	1	0
0	2	0,00	0	
0	0	0,00	0	0
0	1	0,00	0	3
1	1	0,50	2	
2	2	0,00	1	3
1	1	1,00	0	
0	1	1,50	1	3
0	1	0,00	1	16
2	2	0,00	6	0
0	4	0,00	0	0
2	3	1,50	1	0
1	3	1,00	2	7
2	4	2,00	4	0
0	1	0,00	1	6
2	1	0,00	3	1
2	1	1,00	1	3
1	2	0,00	1	
2	2	0,50	2	6
0	1	0,00	0	
1	1	1,00	1	
2	2	0,00	3	
0	1	1,50	0	
1	1	1,50	1	4
2	1	0,00	2	10
2	1	2,50	6	12
2	2	0,00	1	
2	1	0,00	0	4
2	0	0,50	0	
2	3	1,00	1	4
2	1	1,50	1	
2	2	0,50	1	4
2	2	0,50	1	
2	1	0,50	5	
1	1	2,00	2	2

2	4	3,00	5	5
2	3	2,50	3	
2	3	1,00	0	0
2	3	0,00	0	
2	3	2,50	2	0
2	3	1,50	4	
2	3	2,00	6	2
2	2	3,00	6	3
2	2	3,00	6	1
2	3	2,50	6	2
2	4	2,00	6	3
2	4	2,50	6	1
1	4	2,00	6	3
2	2	2,50	4	2
2	3	1,00	5	2
2	2	2,00	6	1
2	2	2,00	4	1
2	3	2,00	6	1
2	2	2,00	5	1
2	3	2,50	5	3
2	2	1,00	6	4
2	3	1,00	5	3
2	3	3,00	5	2
1	3	2,50	5	1
2	3	2,50	5	1
2	2	3,00	5	0
2	2	3,00	4	0
2	3	2,00	5	2
2	1	2,50	4	19
2	2	2,50	6	23
2	2	1,00	1	13
2	1	2,50	6	14
